# Supplementary material for: Antifungal Activities of Multi-Halogenated Indoles Against Drug-Resistant Candida Species
Source: Int J Mol Sci. 2025 Nov 7;26(22):10836. doi: 10.3390/ijms262210836 (PMC12652874; doi:10.3390/ijms262210836)
Supplement: Supplementary file 1 [file ijms-26-10836-s001.zip › ijms-3965689-supplementary.pdf]

International Journal of
Molecular Sciences

MDPI

Type of the Paper (Article, Review, Communication, etc.)

Antifungal activities of multi-halogenated indoles against drug-resistant *Candida* species

HyeonWoo Jeong, Bharath Reddy Boya, Yong-Guy Kim, Jin-Hyung Lee *, and Jintae Lee *

[1] School of Chemical Engineering, Yeungnam University, Gyeongsan, 38541, Republic of Korea; wawdgud@yu.ac.kr (H.-W.J.); bharathreddy2696@yu.ac.kr (B.R.B.); yongguy7@ynu.ac.kr (Y.-G.K.)

* Correspondence: jinhlee@ynu.ac.kr (J.-H.L.); jtlee@ynu.ac.kr (J.L.); Tel.: +82-53-810-3812 (J.-H.L.); Tel.: +82-53-810-2533 (J.L.)

1. Supplementary

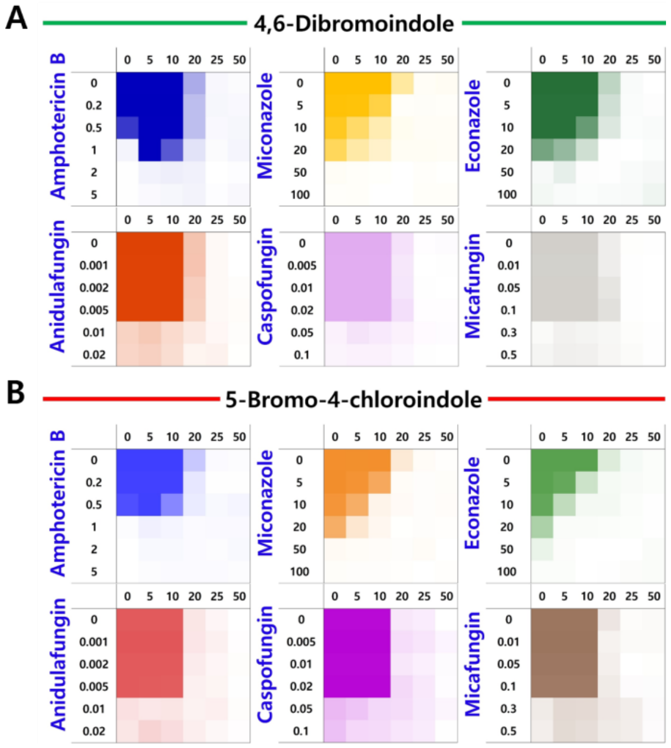

Figure S1. Combinatorial effects with conventional antifungals. Combinatorial inhibition of biofilm formation of C. albicans DAY 185 between 4,6-dibromoindole (A), 5-bromo-4-chloroindole (B) and six conventional antifungals – amphotericin B, miconazole, econazole, anidulafungin, caspofungin, and micafungin.

Table S1. Antifungal activity of multi-halogenated indoles. MICs were determined after 24 h culture in 96-well plates.

No.	Name	Structure	MIC (μg/mL)	CAS Number
1	Indole	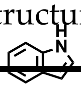	750	120-72-9
2	5-Iodoindole	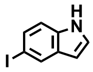	75	16066-91-4
3	3-Bromo-6-chloro-5-azaindole	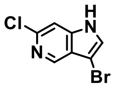	>400	1000341-61-6
4	3-Bromo-6-chloro-7-azaindole	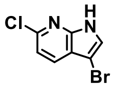	>400	1190321-08-4
5	4-Bromo-3-chloroindazole	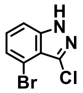	50	1000343-46-3
6	5-Bromo-3-chloroindazole	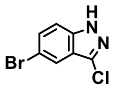	>400	36760-19-7
7	6-Bromo-3-chloroindazole	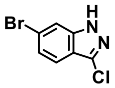	200	885271-78-3
8	6-Bromo-5-chloroindazole	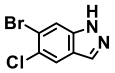	>400	1305208-02-9
9	7-Bromo-3-chloroindazole	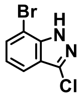	200	885271-75-0
10	1-Acetyl-2,3-dihydroindole-5-sulfonyl chloride	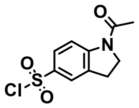	>400	52206-05-0
11	4-Bromo-5-fluoroindole	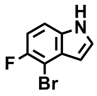	50	1227493-96-0
12	4-Bromo-6-chloroindole	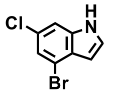	50	885519-23-3

13	4-Bromo-6-fluoroindole	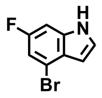	50	885520-70-7
14	4-Bromo-7-chloroindole	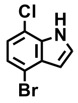	50	126811-30-1
15	4-Bromo-7-fluoroindole	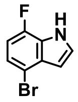	100	883500-66-1
16	4-Bromo-5,7-difluoroindole	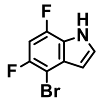	200	1381878-59-6
17	4,5-Dichloroindole	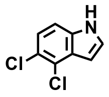	50	122509-73-3
18	4,6-Dibromoindole	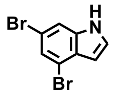	25	99910-50-6
19	4,6-Dichloroindole	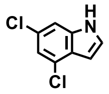	50	101495-18-5
20	4,6-Dichloroindole-2,3-dione	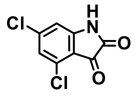	100	18711-15-4
21	4,6-Difluoroindole	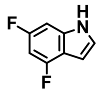	100	199526-97-1
22	4,7-Dibromoindole	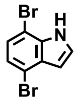	50	126811-31-2
23	4,7-Dichloroisatin	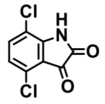	400	18711-13-2

24	5-Bromo-4-chloroindole	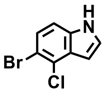	25	217656-69-4
25	5-Bromo-4-fluoroindole	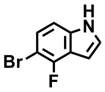	50	344790-96-1
26	5-Bromo-6-chloroindole	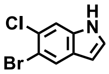	50	122531-09-3
27	5-Bromo-6-fluoroindole	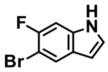	200	434960-42-6
28	5-Bromo-6-chloro-2,3-dihy-droindole	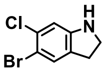	>400	1368146-95-5
29	5-Bromo-7-chloroindole	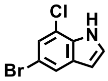	50	180623-89-6
30	5-Bromo-7-fluoroindole	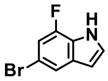	200	883500-73-0
31	5-Chloro-6-fluoroindole	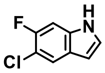	100	169674-57-1
32	5,6-Dibromoindole	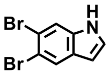	50	854923-38-9
33	5,6-Dichloroindole	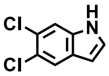	50	121859-57-2
34	5,6-Dichloroindole-2,3-dione	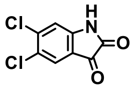	>400	1677-48-1
35	5,6-Dichloroindolin-2-one	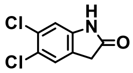	400	71293-59-9
36	5,6-Difluoroindole	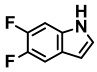	400	169674-01-5
37	5,7-Dibromoindole-2,3-dione	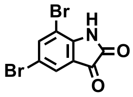	50	6374-91-0
38	5,7-Dibromoindole	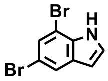	50	36132-08-8

39	5,7-Dichloroindole	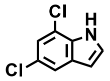	50	4792-72-7
40	6-Bromo-4-iodoindole	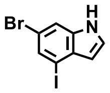	50	885519-17-5
41	6-Bromo-4-fluoroindole	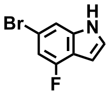	50	885520-59-2
42	6-Bromo-5-chloroindole	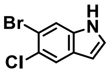	400	1191028-50-8
43	6-Bromo-5-fluoroindole	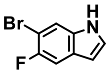	200	259860-08-7
44	6-Chloro-5-fluoroindole	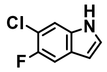	100	122509-72-2
45	6-Chloro-7-fluoroindole	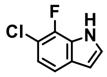	200	259860-04-3
46	6-Fluoro-4-iodoindole	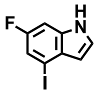	50	885520-49-0
47	6-Fluoro-5-iodoindole	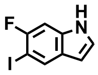	50	908600-77-1
48	7-Bromo-4-chloroindole	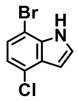	200	126811-29-8
49	7-bromo-4-fluoroindole	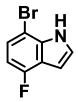	200	292636-09-0
50	7-Bromo-5-chloroindole	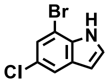	50	292636-08-9
51	7-Bromo-5-fluoroindole	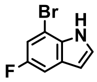	200	408355-23-7
52	7-Chloro-5-fluoroindole	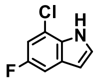	200	259860-01-0

Table S2. Evaluation statistics of the PLS-factor QSAR models.

PLS factor	SD	R^2	Stability	F	P	RMSE	Q^2	Pearson-r
1	0.3991	0.2573	0.825	10.7	0.00259	0.46	0.2108	0.546
2	0.353	0.4376	0.575	11.7	0.000178	0.36	0.499	0.7146
3	0.3175	0.5602	0.389	12.3	2.27E-05	0.36	0.5182	0.751
4	0.2981	0.6256	0.391	11.7	1.04E-05	0.29	0.6726	0.8318
